# Analysis of salivary fluid and chemosensory functions in patients treated for primary malignant brain tumors

**DOI:** 10.1007/s00784-014-1211-8

**Published:** 2014-03-05

**Authors:** Susan Mirlohi, Susan E. Duncan, Michele Harmon, Doug Case, Glenn Lesser, Andrea M. Dietrich

**Affiliations:** 1 Department of Civil and Environmental Engineering, Virginia Tech, Blacksburg, VA USA; 2 Department of Food Science and Technology, Virginia Tech, 413 Durham Hall, 0246, Blacksburg, VA 24061-0246 USA; 3 Department of Internal Medicine-Section of Hematology and Oncology, Wake Forest School of Medicine, Winston-Salem, NC USA; 4 Division of Public Health Sciences, Wake Forest School of Medicine, Winston-Salem, NC USA

**Keywords:** Metallic flavor, Oral lipid oxidation, Cancer, Brain tumor, Chemotherapy, Saliva, Taste and smell

## Abstract

**Objectives:**

The frequency and causes of chemosensory (taste and smell) disorders in cancer patients remain under-reported. This study examined the impact of cancer therapy on taste/smell functions and salivary constituents in brain tumor patients.

**Materials and methods:**

Twenty-two newly diagnosed patients with primary malignant gliomas underwent 6 weeks of combined modality treatment (CMD) with radiation and temozolomide followed by six monthly cycles of temozolomide. Chemosensory functions were assessed at 0, 3, 6, 10, 18, and 30 weeks with paired samples of saliva collected before and after an oral rinse with ferrous-spiked water. Iron (Fe)-induced oxidative stress was measured by salivary lipid oxidation (SLO); salivary proteins, electrolytes, and metals were determined. Parallel salivary analyses were performed on 22 healthy subjects.

**Results:**

Chemosensory complaints of cancer patients increased significantly during treatment (*p* = 0.04) except at 30 weeks. Fe-induced SLO increased at 10 and 18 weeks. When compared with healthy subjects, SLO, total protein, Na, K, Cu, P, S, and Mg levels, as averaged across all times, were significantly higher (*p* < 0.05), whereas salivary Zn, Fe, and oral pH levels were significantly lower in cancer patients (*p* < 0.05). Neither time nor treatment had a significant impact on these salivary parameters in cancer patients.

**Conclusions:**

Impact of CMT treatment on chemosensory functions can range from minimal to moderate impairment. Analysis of SLO, metals, and total protein do not provide for reliable measures of chemosensory dysfunctions over time.

**Clinical relevance:**

Taste and smell functions are relevant in health and diseases; study of salivary constituents may provide clues on the causes of their dysfunctions.

## Introduction

Chemosensory (taste and smell) functions are critical aspects of human physiology. They provide us with the pleasures of experiencing flavors and aromas in foods and beverages and the smells of fragrances. They also protect us from the dangers of ingested or inhaled toxins. Thus, disorders of taste and smell functions can have important health implications as well as debilitating effects on our quality of life. Factors associated with taste and smell disorders include aging, environmental exposure, nasal congestion and allergies, prescription medication, head trauma, neurological diseases such as Alzheimer’s, and cancer [1–6].

In cancer patients, taste and smell disorders have been associated with the disease itself and/or the effects of chemo- and radiation therapies [7]. Taste disorders have been described as “nauseating” or “unpleasant” by cancer patients of all ages, including pediatric patients [8–10]. These issues have broad impacts on the quality of life for these patients and often lead to other implications such as reduced oral intake and nutritional deficiency, weight loss, and depression [11–13]. While these problems are significant, there are only a limited number of studies exploring their causes and possible treatments.

Among taste disorders, perception of a persisting metallic and bitter taste or aftertaste in the mouth is the most common disorder described by cancer patients [14]. Sensory perception of metallic off-flavors from ingested foods and beverages is caused by the release of retronasal odors associated with volatile by-products of metal-oxidized lipids within the oral cavity. Metal-induced lipid oxidation also occurs in the oral cavity of healthy human subjects when potable water spiked with copper (Cu) and iron (Fe) is consumed [15–18]. On the human skin surface, lipid oxidation has been linked to the production of “metallic” odor compounds produced when a metallic object such as a key or a penny is held in the hand [19]. Within the oral cavity, polyunsaturated fatty acids in the oral mucosal cell membranes or the salivary fluid lipids can be oxidized to form by-products of lipid oxidation [20]. In cancer patients, occurrence of metallic and bitter taste sensations has been associated with exposure to low levels of irradiation and chemotherapy [21–23]. Release of chemotherapy drugs into the salivary fluid and interaction with taste buds can cause impairments by direct contact with taste receptor cells. Additionally, damage to sensory nerves such as the chorda tympani can cause disturbances in taste functions [24]. As side effects of treatment, taste and smell abnormalities are most commonly reported in head and neck cancer cases [25, 26], which include cancers of the organs in the regions of the throat, nasal/oral cavities, and salivary glands [27]. Abnormal taste and smell symptoms are anticipated in other types of cancers where sensory organs are affected by the field of radiation treatment, as is the case for primary malignant brain tumors or gliomas. Currently, the most common treatment for such tumors is surgery, followed by concurrent radiation and chemotherapy—typically an orally administered drug called temozolomide (Temodar). Temozolomide is then given on monthly cycles for 6–12 months as maintenance therapy [28].

The Central Brain Tumor Registry of the US has reported that approximately 24,000 new primary malignant brain tumors are diagnosed in the USA each year [29]. Although most prevalent among the elderly (greater than 50 years of age), brain tumors occur in all ages and are the second most frequent malignancy of childhood after leukemia [29]. Currently, no prospective data are available on the incidence, severity or duration of the taste and smell abnormalities experienced by brain tumor patients undergoing concurrent radiation and chemotherapy. Anecdotally, a large number of these patients develop dramatic alterations in perception of food flavor, texture, quality, and the sensation of a lingering metallic taste within several weeks of beginning radiation therapy, with or without concurrent chemotherapy [30]. These alterations lead to food aversion, weight loss, and a profoundly decreased quality of life [2, 12]. They may last for up to several months after the radiation has been completed and, on occasion, normal taste and odor sensation never returns [2]. The objectives of this study were (1) to quantify taste and smell abnormalities in a small cohort of newly diagnosed primary glioma patients treated with combined modality treatment (CMT); (2) to assess the etiologic role of salivary oxidative stress and constituents, as cause of the observed sensory changes; and (3) to compare the salivary constituents in the cancer patients with healthy subjects. It is hoped that the findings from this research will provide baseline data and lead to future trials utilizing an intervention to prevent or treat taste and smell disturbances in similar patient populations.

## Materials and methods

### Human subjects

This study was approved by the Institutional Review Board at Virginia Tech and the Wake-Forest School of Medicine. Twenty-two cancer patients (ten females), with ages ranging from 20 to 79 years (median age, 60), were recruited from the brain tumor clinic at the Comprehensive Cancer Center of Wake Forest University. Eligibility criteria included age greater than 18 years, a newly diagnosed primary malignant brain tumor, anticipated combined modality therapy, and an expected survival of at least 6 months. Ineligible patients included those with an extreme dry mouth syndrome that prevented them from producing adequate amounts of saliva (about 2 mL in 15–20 min), known HIV-positive status, and with any of the following conditions: untreated gastroesophageal reflux disease; uncontrolled diabetes; active oral infections including thrush; or evidence of active mucositis. Additionally, 22 healthy subjects (14 females), with ages ranging from 21 to 82 years (median age, 58), were recruited from the community, students, faculty, and staff of Virginia Tech and Blacksburg, Virginia. Healthy subjects were required to have no chronic oral or general health problems, be nonsmokers, and not pregnant. All subjects read and signed an informed consent form in accordance with the approved protocols.

### Cancer treatment plan

**Fig. 1:**
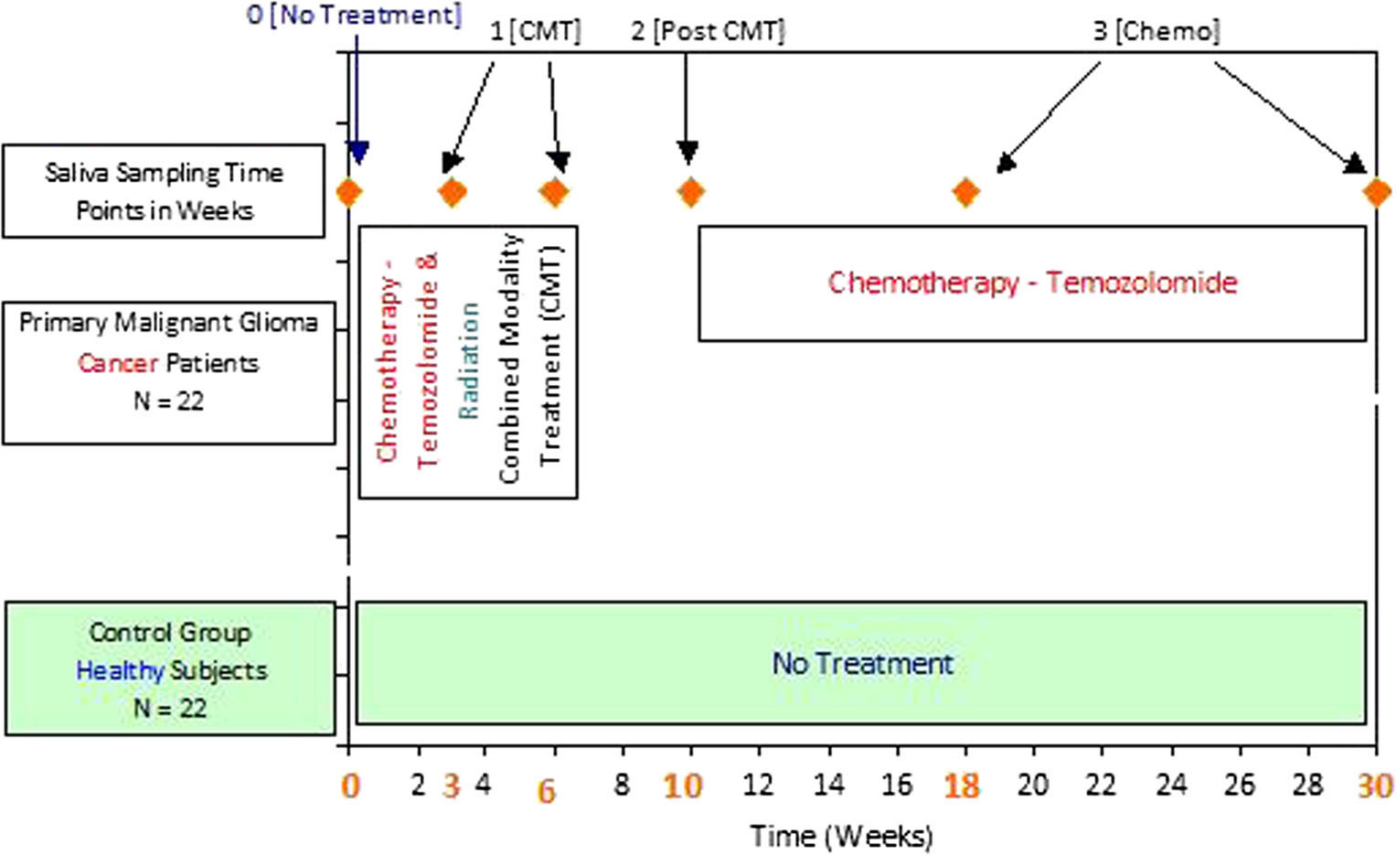
Saliva collection and cancer treatment regimen. Study subjects donated saliva at time 0, 3, 6, 10, 18, and 30 weeks. For cancer patients, time 0 represents the baseline (prior to the start of cancer treatment). Treatment designation for data plotting and analysis purposes include: 0 (*no treatment* or none), representing baseline or 0 time; 1 (*CMT*) representing 3- and 6-week times; 2 (*post-CMT*), representing 10-week time; and 3 (*Chemo*), representing 18- and 30-week times. *CMT* combined modality treatment

Cancer patients received a CMT, composed of standard radiation therapy with concurrent temozolomide, over 6 weeks followed by adjuvant temozolomide given for 5 days each month for an additional 6 months. Patients underwent an initial, baseline saliva analysis and chemosensory assessment prior to beginning CMT. Repeat analyses were performed following 3, 6, 10, 18, and 30 weeks of treatment. For healthy subjects, saliva analysis was performed at 0 (baseline), 3, 6, 10, 18, and 30 weeks. A schematic of cancer treatment regimen and saliva collection is depicted in Fig. 1. Among cancer patients, 17 subjects (seven females) had baseline and CMT (3–6 weeks) or post-CMT (10 weeks) measurements and were included in the data analysis. Most subjects provided data through 10 weeks, but not all patients were able to provide data at each time point and four dropped out after 10 weeks due to their disease progress. The numbers of subjects at 0, 3, 6, 10, 18, and 30 weeks were 17, 15, 15, 16, 12, and 12, respectively. Data were complete for the healthy subjects. One patient was a current smoker, but stopped smoking at the time of diagnosis; 5 patients were previous smokers who had stopped 5 to 30 years earlier, and the remaining patients were nonsmokers.

### Saliva collection and analysis

At baseline and each time point, saliva samples were collected from subjects (who had not consumed food or beverages and had not smoked for at least one hour prior to testing) as follows. First, subjects rinsed their mouth using purified water (Aquafina®, which is treated by reverse osmosis and has chemical properties similar to distilled water). After a 1-minute rest, they sipped 2 mL of purified water, as the control sample, then, swished it around their mouth for 20 sec., and, without swallowing, expectorated saliva into a clean sample collection tube until approximately 4 mL of saliva (control) was collected. After a short rest period, subjects sipped 2 mL of ferrous sulfate (Sigma-Aldrich, PA, CAS # 13463-43-g) solution (at 10 ± 1 mg/L ferrous), swished the solution around their mouth, and expectorated the second (ferrous) 4 mL saliva sample. Subjects were asked to put on nose clips before sipping the metal salt solution to help evaluate the retronasal component of the metallic flavor sensation. Subjects’ oral pH was measured using a pH indicator strip (Cen-Med/Fisher M95883) after sampling the control solution and again after sampling the ferrous salt solution. Saliva samples were frozen immediately and stored at −50 ºC for up to 3 months until analysis. Saliva analysis included the measurement of Fe-induced salivary oxidative stress response as thiobarbituric acid reactive substances (TBARS), total protein, and specific metals, nonmetals, and electrolytes.

### Fe-induced salivary oxidative stress response

TBARS method was used for the measurement of salivary oxidative stress by lipid oxidation. The method was modified from Spanier’s to work with liquid samples and to enhance readings at low concentrations [31]. For TBARS analysis, 1 mL of saliva sample and known concentrations of malondialdehyde (MDA) standard were mixed with 2 mL of thiobarbituric acid (TBA) solution and digested for 60 min at 95 °C in a water bath. Then, the samples were immediately cooled in an ice bath, mixed with 2 mL of *n*-butanol/pyridine mixture (at 15:1 ratio), and centrifuged for 15 min. at a speed of 3,000×*g*. The absorbance of the supernatant was measured with a spectrophotometer at 532 nm for the samples and standard curve. The concentration of TBARS was obtained from the MDA standard curve and absorbance values. The dilution effect was taken into consideration in the calculations. As TBARS is a nonspecific indicator of MDA formation, one of several by-products of lipid oxidation, the results were reported in μM units of TBARS rather than MDA.

For the purpose of data analysis and as an indicator for potentially heightened sensitivity to metallic flavor, Fe-induced oxidative stress response was defined as the arithmetic difference between salivary lipid oxidation (SLO) measured in saliva before and after the oral rinse with ferrous-spiked water.

### Salivary protein

As the protein content of saliva can vary among individuals, to report the TBARS results more accurately, the total protein content of the saliva of the patients, as well as the healthy individuals, was measured and the results were reported as μM TBARS produced per gram of protein. Total protein content of saliva was analyzed using the Bradford assay [32]. A standard curve was obtained by using bovine serum albumin at 1 mg/mL concentration. To perform the analysis, 1 mL of Bradford reagent was mixed with 8 to 20 μL of saliva. The samples were vortexed and 100 μL of the mixture was transferred to the well plates and read by spectrophotometer at 595 nm. Duplicates of each sample were analyzed, and the total protein content of the saliva was determined from the standard curve.

### Salivary metals, nonmetals, and electrolytes

Saliva samples were first thawed at room temperature. Then, they were diluted with deionized water at 1:10 ratio and digested with trace metals grade nitric acid at 90 °C for 45 min.; hydrogen peroxide was added and the solution was heated to 130 °C until the mixture became clear as described by the US Environmental Protection Agency method 3050B [33]. After digestion, the samples were adjusted back to their original dilution volume and acidified with 2 % trace metals grade nitric acid prior to being analyzed by emission spectroscopy using Inductively Coupled Plasma technique [33].

### Chemosensory assessment

There are no standard tools specifically designed to assess taste and smell in cancer patients. Thus, as an exploratory endpoint, self-perceived taste and smell functions were assessed using a validated questionnaire that has been used to evaluate chemosensory functions in AIDS patients [34]. As part of the questionnaire, patients were asked to rate their individual taste and smell abnormalities as “insignificant,” “mild,” “moderate,” “severe,” or “incapacitating.” The tool yields a taste complaint score (0–10) based on the subjects’ responses to nine questions addressing changes to the sense of taste since the start of their cancer treatment. The questionnaire was completed prior to the start of treatment (baseline) and at 3, 6, 10, 18, and 30-week time points following the baseline. For the taste complaint portion of the questionnaire, there were nine questions and scores could vary from 0 to 10. To score the questionnaire, one point was added for each of four questions when the subject indicated an abnormal sensitivity to sweet, sour, salty or bitter. Four additional questions and a possible four points pertained to complaints about specific attributes of taste: (1) foods tasting differently, (2) drugs affecting taste, (3) bad taste in mouth, or (4) a change in the sense of taste. The ninth taste question addressed rating the severity of abnormality for the overall sense of taste. One point was added if the subject rated their overall taste abnormality as “mild” or “moderate”; two points were added if a rating of “severe” or “incapacitating” was reported. Similarly, a smell complaint score (0–6) was generated by adding one point for a positive response to each of five questions addressing self-perceived changes to the sense of smell. One additional point was assigned to the total smell complaint score, if a severity rating of “severe” or “incapacitating” was reported for the smell abnormality. The total chemosensory complaint score (CSCS; score range, 0–16), was calculated by adding the taste and smell complaint scores.

### Data analysis

To assess the significance of the pre/post-rinse differences and the significance of the changes in Fe-induced SLO (delta SLO) over time and treatment periods, repeated measures analysis of variance (ANOVA), which assumes missing data are missing at random and depend on previous measures of the outcome, was used separately for the cancer patients and the healthy subjects. The correlation structure used to model the within patient correlations over time was chosen based on the Bayesian Information Criterion statistic. For treatment periods, data grouping consisted of delta SLO responses at baseline (time 0), during CMT (3- and 6-week time points; averaged within a person across those times), post-CMT (10-week time point), and during adjuvant monthly temozolomide (18- and 30-week time points; averaged within a person across those times). Subjects with missing data at one of the two time periods were included, and the one value was used at the average. Comparisons among baseline, CMT, and the early post-CMT periods included at least 16 of the 17 patients. The later post-CMT period (18 or 30 weeks) included 13 patients.

Delta SLO was the arithmetic difference between the measured salivary TBARS before (control) and after (ferrous) oral rinse with ferrous-spiked water. For the salivary parameters, comparative analyses were performed on the mean responses (averaged within subjects across time) between the healthy subjects and cancer patients using Student’s *t* test with or without a Satterthwaite correction, depending on the equality of variances in the two groups. For chemosensory assessment in cancer patients, repeated measures models, as described above, were used to assess the significance of time and treatment on self-reported taste and smell abnormalities as quantified by the CSC scores. Statistical software programs, JMP 9.0 and SAS (SAS, Cary, NC), were used for the data analyses. For all analyses, *p* values less than 0.05 were considered statistically significant.

## Results

### Fe-induced salivary oxidative stress response

SLO levels before the oral rinse with ferrous-spiked water did not change significantly over time for either the cancer patients or the healthy subjects. Mean levels and standards deviations (mean ± SD), in micromolars TBARS per gram of protein, ranging from 0.47 ± 0.45 to 1.06 ± 1.22 for the cancer patients (*p* = 0.14) and from 0.27 ± 0.21 to 0.43 ± 0.46 in healthy subjects (*p* = 0.73). Mean levels were higher for cancer patients at every time, and on average, the mean level in cancer patients was significantly greater than that of healthy subjects (0.80 ± 0.71 vs. 0.35 ± 0.14, *p* = 0.020). Similarly, SLO levels after the ferrous rinse did not change significantly over time in either the cancer patients (*p* = 0.059) or the healthy subjects (*p* = 0.587), although the mean increase of over 5 units from 6 to 10 weeks in cancer patients was interesting. On average, the SLO levels after the ferrous rinse were nonsignificantly higher for cancer patients compared with healthy subjects (3.01 ± 4.06 vs. 1.14 ± 0.46, *p* = 0.078). The between-subject variability was surprisingly high at 10- and 18-week time points, with respective means and standard deviations of 6.60 ± 9.83 and 6.60 ± 12.83.

Delta SLO represents the Fe-induced SLO response calculated by the arithmetic difference between the measured salivary TBARS before (control) and after (ferrous) oral rinse with ferrous-spiked water. Changes in the delta SLO levels over time as well as during cancer treatment were highly variable for the cancer patients, with mean levels ranging from 0.22 to 5.91 μM TBARS/g protein; changes in the delta SLO over time were much more consistent for the healthy subjects with mean delta SLO ranging from 0.67 to 0.88 μM TBARS/g protein. The change in delta SLO over time was not statistically significant for either the healthy subjects (*p* = 0.98) or the cancer patients (*p* = 0.11). Likewise, when mean delta SLO responses for the cancer patients were evaluated by treatment phase, the differences were non-significant (*p* = 0.16).

At each time point, mean SLO levels typically increased after oral rinse with ferrous-spiked water in both cancer patients and healthy subjects. The increase in mean SLO after ferrous rinse was consistently significant in healthy subjects (*p* ≤ 0.005 for all times), while the increase for cancer patients was significant at all times (*p* < 0.029) except 6 weeks (*p* = 0.410). The increase in SLO response after oral rinse with ferrous-spiked water corresponded with perception of a strong metallic sensation in the mouth as verbally described by both subject groups. The metallic sensation was absent or barely perceived when subjects’ nostrils were closed using nose clips, indicative of a retronasal perception phenomenon as described by other researchers [17, 35].

### Chemosensory assessment

**Fig. 2:**
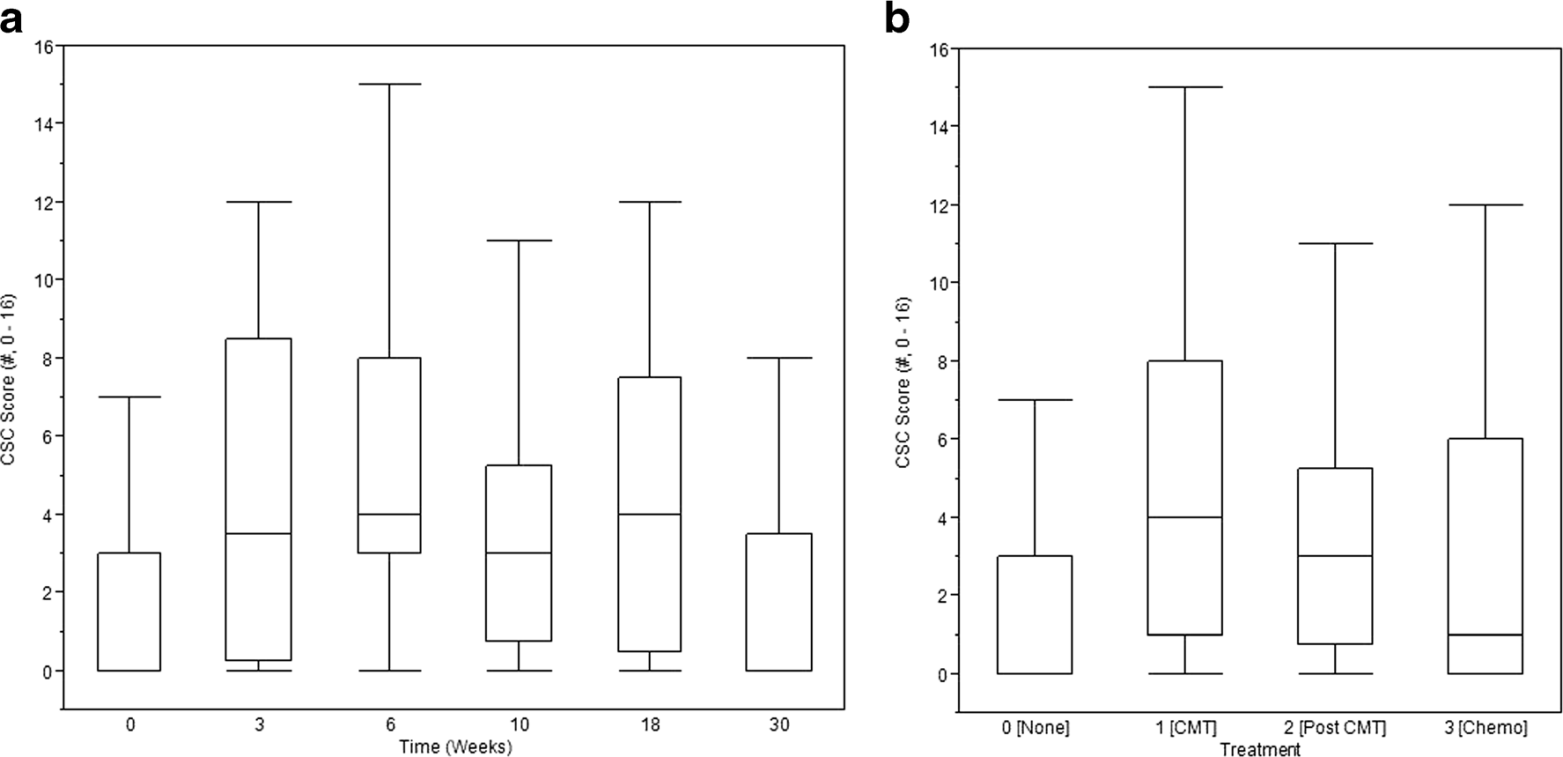
Chemosensory complaint scores (*CSC*) for cancer patients (*N* = 17) as measured by self-reported taste and smell questionnaire. The plotted data represent CSC scores over time (**a**) and the course of the cancer treatment (**b**)

Self-reported taste and smell abnormalities for individual cancer patients, as assessed by the CSC score scale of 0 to 16 (none to maximal impairment), ranged from 0 to 15. CSC scores for cancer patients as plotted against time in weeks and treatment phase are displayed in Fig. 2. Changes in CSC scores, mean ± SD, were significant over time from 3 to 30 weeks (1.6 ± 2.4, 4.6 ± 4.1, 5.1 ± 4.1, 3.6 ± 3.4, 4.4 ± 4.1, and 2.3 ± 4.2, *p* = 0.04; Fig. 2a) as well as treatment (*p* = 0.03; Fig. 2b). For the time effect, the significant mean differences were between the baseline (time 0) and all the times, except 30 weeks. At baseline and each corresponding time point, the numbers of patients reporting taste complaints were 7, 9, 12, 9, 6, and 4. The taste complaint scores ranged from a minimum of 0 and mean of 1.2 ± 1.9 at baseline to a maximum of 10 and mean of 3.5 ± 2.7 at 6-week time point. The smell complaint scores ranged from a minimum of 0 and mean of 0.5 ± 1.0 at baseline to a maximum of 5 and mean of 1.7 ± 1.9 at 10 weeks and mean of 1.9 ± 2.1 at 18 weeks time points. A majority of the patients (14 out of 17) reported having both taste and smell complaints at some point during the treatment while three patients reported having only taste complaints.

### Salivary protein

**Fig. 3:**
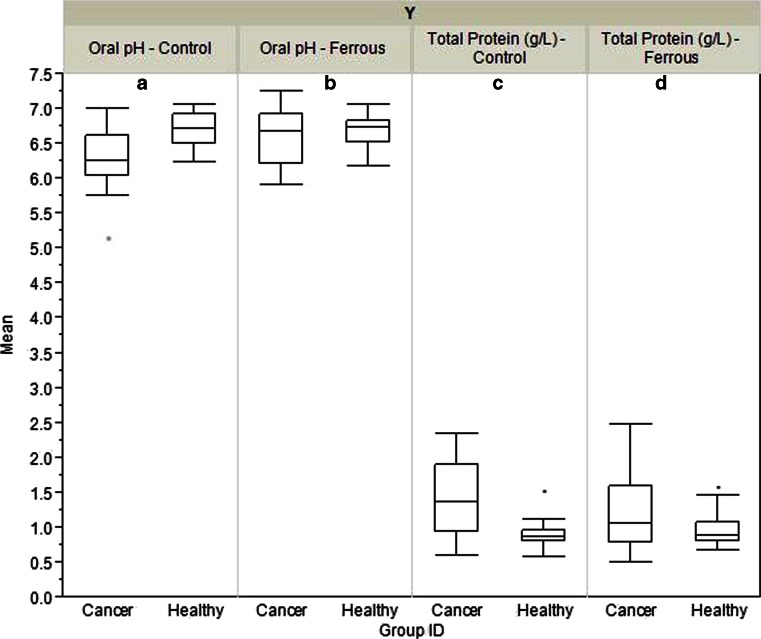
Comparisons of mean levels of oral pH (*a*, *b*) and total salivary protein levels (*c*, *d*) in cancer patients (*N* = 17) and healthy subjects (*N* = 22)

The changes over time in mean total salivary protein levels [before (control) and after the ferrous rinse] were not statistically significant for healthy subjects or cancer patients. Also, the changes over treatment phases for the cancer patients were not significant (*p* > 0.05 for all tests). Salivary protein levels, as averaged across all times, are shown in Fig. 3(c (control), d (ferrous)). The mean control salivary protein levels in g/L, averaged across time within a subject, were significantly greater for cancer patients compared with healthy controls (1.42 ± 0.54 vs. 0.89 ± 0.19; *p* = 0.001). The mean ferrous salivary protein level in grams per liter was also higher for cancer patients, though not significantly so (1.22 ± 0.52 vs. 0.96 ± 0.24; *p* = 0.08). The mean total salivary protein levels decreased significantly pre- to post-ferrous rinse for cancer patients (*p* = 0.005) but not for healthy controls (*p* = 0.247).

### Measurements of oral pH

Control and ferrous oral pH levels showed no significant changes over time in either group. Control sample oral pH levels (averaged across time within subjects) were significantly lower in cancer patients than in healthy subjects (6.29 ± 0.46 vs. 6.70 ± 0.23; *p* = 0.003; Fig. 3(a)). Ferrous sample pH levels did not differ significantly between groups (6.58 ± 0.44 vs. 6.69 ± 0.23; *p* = 0.38; Fig. 3(b)). The change in oral pH levels (comparing saliva samples before and after ferrous rinse) was statistically significant for cancer patients (*p* = 0.04) but not for healthy subjects (*p* = 0.61).

### Salivary metals, nonmetals, and electrolytes

In cancer patients, the concentrations of salivary constituents showed no statistically significant changes over time (*p* = 0.06 to 0.81) or by treatment (*p* = 0.12 to 0.93). For the healthy subjects the mean change in the salivary constituents was significant over time only for Cu (*p* = 0.005), zinc (Zn; *p* = 0.02), and Fe (*p* < 0.0001). While copper levels showed no clear pattern over time, Zn and Fe levels tended to decrease over time. Although changes in salivary Zn and Fe between subjects and over time could be associated with nutritional status of individuals [36], reasons for this declining trend were not explored. Except for chloride (Cl) and Ca, the mean levels of all constituents (averaged within patients across time) differed significantly between cancer patients and healthy subjects (Table 1). Mean salivary levels of sodium (Na), magnesium (Mg), phosphorus (P), sulfur (S), potassium (K), and Cu were greater for cancer patients than for healthy subjects while Zn and Fe levels (both before and after ferrous rinse) were significantly lower for cancer patients than for healthy subjects.

**Table 1:** Comparison of salivary electrolytes, metals, and nonmetals in cancer patients and healthy subjects

Salivary parameter	Measured concentration (mg/L: mean ± standard deviation (95 % CI))		Means comparison^a^
	Cancer group	Healthy group	*p* value
Elements (total)			
Na	211.28 ± 49.02 (186.08–236.48)	169.23 ± 41.11 (151.00–187.46)	0.006
Mg	8.69 ± 0.55 (7.54–9.85)	5.49 ± 1.35 (4.89–6.09)	<0.0001
P	222.67 ± 59.21 (192.23–253.11)	166.69 ± 47.45 (145.65–187.73)	0.002
S	346.33 ± 87.86 (301.16–391.50)	237.98 ± 44.61 (218.20–257.76)	<0.0001
Cl	621.57 ± 170.77 (533.76–709.37)	553.20 ± 98.24 (509.64–596.76)	0.12
K	1088.40 ± 245.74 (962.02–1214.70)	776.28 ± 130.81 (718.28–834.27)	<0.0001
Ca	74.46 ± 19.04 (64.67–84.24)	67.04 ± 13.08 (61.24–72.85)	0.16
Cu	0.09 ± 0.04 (0.07–0.11)	0.06 ± 0.04 (0.04–0.08)	0.05
Zn	0.41 ± 0.17 (0.32–0.49)	0.54 ± 0.12 (0.49–0.60)	0.005
Fe (before Fe^+2^ rinse)	0.49 ± 0.12 (0.43–0.55)	1.28 ± 0.21 (1.19–1.37)	<0.0001
Fe (after Fe^+2^ rinse)	1.96 ± 0.39 (1.76–2.17)	3.68 ± 0.23 (3.58–3.78)	<0.0001

^a^Comparison of the overall mean of the salivary parameters between the healthy subjects (*n* = 22) and cancer patients (*n* = 17), as averaged across all times, within subjects

## Discussion

Our results show that in this group of cancer patients, the impact of CMT on impairment of chemosensory functions was significant; however, chemosensory impairment, as assessed over the course of treatment, did not necessarily coincide with Fe-induced salivary oxidative stress response and other measured saliva constituents. Conversely, the significant differences in multiple salivary parameters between cancer patients and healthy subjects can provide clues on the causes and/or identify potential biomarkers of chemosensory disorders, as highlighted in Table 2. The findings are further discussed in the proceeding sections.

**Table 2:** Comparison of salivary electrolytes, metals, and nonmetals in cancer patients and healthy subjects

Salivary parameter^a^	Within-subject effects		Cancer vs. healthy (across all times)	Potential relevance to metallic taste disorders
	Time	Cancer treatment		
Iron-induced SLO (μM TBARS/g protein)	NS	NS	NS cancer > healthy	Heightened metallic flavor sensitivity may correspond to higher SLO
Chemosensory response (as CSCS)	Significant (*p* = 0.04)	Significant (*p* = 0.03)	–	Heightened sensitivity corresponds to higher CSCS
Total salivary protein	NS	NS	Significant cancer > healthy (*p* = 0.001)	Specific proteins can influence taste perception through metal binding
Salivary elements (total): Na, K, and Mg	NS	NS	Significant cancer > healthy (*p* ≤ 0.006)	Concentrations influenced by salivary flow rate, which can in turn impact taste perception
Ca and Cl	NS	NS	NS	Concentrations influenced by salivary flow rate
P and S	NS	NS	Significant cancer > healthy (*p* ≤ 0.002)	Unknown
Fe	NS	NS	Significant cancer < healthy (*p* < 0.0001)	Fe retained in the oral cavity, bound to proteins and/or enamel surfaces
Cu	NS	NS	Significant cancer > healthy (*p* = 0.05)	Fe retained in the oral cavity, bound to proteins and/or enamel surfaces
Zn	NS	NS	Significant cancer < healthy (*p* = 0.005)	Zn deficiency can influence taste functions
Oral pH	NS	NS	Significant cancer < healthy (*p* = 0.003)	Effects on metal speciation and protein binding. Also influenced by salivary flow rate

^a^Salivary parameters measured in the control saliva (prior to oral rinse with ferrous-spiked water), except for the Fe-induced salivary lipid oxidation, which represents the iron-induced SLO response calculated by the arithmetic difference between the measured salivary TBARS before (control) and after (ferrous) oral rinse with ferrous-spiked water

*NS* not significant at alpha level (0.05), *CSCS* chemosensory complaint score, SLO salivary lipid oxidation

### Salivary oxidative stress response and chemosensory assessment

The overall mean SLO levels in control saliva samples collected before oral rinse with ferrous-spiked drinking water was significantly higher in cancer patients than in healthy subjects. Elevated oxidative stress response in biological fluids such as serum and saliva is a common occurrence in some disease conditions including cancer [37, 38]. The increase in oxidative stress response is associated with free-radical reactions that cause damage to macromolecular components, such as lipids, proteins, and DNA in cells and tissues [39]. Transition metals, such as Fe, are known for their catalytic effects on free-radical oxidative reactions [40]; therefore, the higher mean SLO responses in cancer patients and healthy subjects is not surprising after oral rinse with ferrous-spiked water. The overall increase in the intensity of Fe-induced oxidative stress response was somewhat consistent with the chemosensory responses as measured by the mean CSC scores, which increased significantly with respect to time and treatment (*p* < 0.05) as compared with the baseline; however, the findings were not uniform. For example, in some cancer patients, the mean CSC scores were parallel to that of the Fe-induced oxidative stress response as measured by delta SLO, whereas in other patients the Fe-induced oxidative stress response did not correspond with the CSC scores. By contrast, prior to the start of cancer treatment (at baseline or time 0), Fe-induced SLO and CSC scores in cancer patients showed a relatively strong linear correlation (*R* ^2^ = 0.84; *p* < 0.0001), no such correlation existed at time points (3 through 30 weeks) during the treatment (*R* ^2^ ranging from 0.0003 to 0.06). This finding indicates that although Fe-induced SLO can be used as an indicator of sensory response to metallic flavor perception in both healthy subjects and cancer patients, it is not a reliable biomarker of chemosensory impairment associated with cancer therapy in glioma patients.

On the persistence of self-reported taste and smell abnormalities as measured by the CSC scores obtained using a validated questionnaire, our results are consistent with previous findings. Namely, a study in patients with breast cancer or gynecological malignancies showed that taste and smell functions, as assessed by sniff sticks and taste strips, declined significantly during chemotherapy, but normal functioning returned 3 months after chemotherapy [41]. In our study, sensory abnormalities occurred and persisted during cancer treatment but returned to the baseline levels 30 weeks after the start of treatment. As a limitation, the quantified taste complaint scores in our study assessed the patients’ self-reported complaints associated with rating of persistent, unpleasant and/or abnormal tastes in the mouth, and the presence or absence of abnormal sensitivity to the taste sensations of salty, sweet, sour, and bitterness. Therefore, the quantified chemosensory complaint scores are not exclusively indicative of metallic flavor complaints. However, the heightened sensitivity to metallic sensation was qualitatively assessed through patients’ verbal descriptors of strongly perceived metallic sensation and the corresponding measure of salivary oral lipid oxidation responses before and after oral rinse with ferrous-spiked water. The strongly metallic flavor was perceived only with the nose open. This is indicative of the patients’ abilities to perceive the metal-induced retronasal smell, as partly reflected by the lower occurrence of self-reported smell complaints relative to taste complaints. A previous study evaluating age-related sensitivities to metallic flavor of Fe in healthy adults associated impaired olfactory functions with a diminished sensitivity to metallic flavor [16].

Another limitation is the small sample size for cancer patients and the fact that not all patients were able to provide data at each time point. No patient had complete data and four patients dropped out of the study after 10 weeks.

### Variations in oral pH and potential influence on taste perception

As presented earlier, our results indicate that the mean oral pH level in cancer patients was significantly lower (*p* < 0.0001) than that of healthy subjects. As pH is an important factor in speciation of metals in aqueous environments, variations in pH could be an implicating factor in taste perception. A previous study on Cu has shown that taste perception is associated with soluble species of Cu and that the particulate form, which generally forms above pH 7, is poorly tasted [42]. In the case of Fe, within the typical pH range of saliva (5.5–8.0), Fe is expected to remain in the dissolved ferrous form [43]. In the presence of salivary proteins, the role of pH can be further complicated. Studies with artificial saliva have shown that the metal-binding capacities of major salivary proteins, mucin and alpha amylase, can decrease or increase based on the metal concentration as well as the salivary pH [44]. Additionally, depending on their isoelectric points in relation to the pH of saliva, different salivary proteins can be influenced to either inhibit or enhance lipid oxidation reactions [45].

### Salivary metals, nonmetals, and electrolytes

As a noninvasive method of biological sample collection, analysis of salivary fluid for metals and electrolytes has been extensively studied in the literature for the purposes of exposure assessment to toxic metals or diagnostics in clinical applications [44, 46]. It is recognized that there are inherently wide variations between and within subjects on salivary parameters due to variations in salivary flow, whereas possibly age and gender could be sources of variations as well [47]. Our research showed that mean levels of salivary electrolytes, metals and nonmetals, specifically, Na, Cl, K, Mg, P, and S were significantly higher in the cancer patients (*p* < 0.05) when compared with the healthy subjects, whereas the mean level of total Zn and Fe was significantly lower in the cancer patients than healthy subjects (*p* < 0.0001).

Zn deficiency has been widely associated with decline in taste acuity, although the findings are conflicting. In a clinical study with a group of head and neck cancer patients [48], it was demonstrated that oral administration of Zn sulfate alleviated taste abnormalities in cancer patients treated with external beam radiation therapy and improved the recovery of their taste acuity after the treatment. Similar findings have been reported in other studies [46], whereas in other cases Zn supplementation failed to prevent taste alterations in head and neck cancer patients undergoing radiotherapy as demonstrated in a placebo-controlled trial [49].

One interesting finding from our study is the consistently significant lower level of Fe in saliva of cancer patients compared with that of healthy subjects (*p* < 0.0001). This observation remained even after the oral rinse with drinking water containing 10 ± 1 mg/L of ferrous sulfate, indicative of significantly lower recovery of Fe in saliva of cancer patients after the oral rinse with ferrous sulfate.

A possible reason for the observed low Fe levels in saliva of cancer patients relative to the healthy subjects could be associated with nutritional state of the patients. Among side effects of chemotherapy, conditions of anemia and Fe deficiency are common and often treated with Fe supplementation or therapeutic agents that boost red blood cells production [50, 51].

As expected, the concentration of total Fe recovered in saliva after oral rinse with ferrous-spiked water was higher than the pre-rinse concentrations; however, the notably low post-rinse recovery of Fe from the saliva of the cancer patients as compared with the healthy subjects is puzzling. In exploring possible reasons for this low recovery, the loss of Fe could be associated with the rapid uptake of Fe by Fe-binding proteins that were not recovered in the expectorated saliva, accidental swallowing of the Fe-spiked oral rinse water, and binding of the protein-complexed Fe to oral cavity tissues and/or teeth surfaces. Dental research has shown that a number of salivary proteins, namely proline-rich proteins, cystatins, statherin, and histatins selectively bind to enamel surfaces and hydroxyapatite [52]. Additionally, radiation therapies targeting tumors in the head and neck region have been shown to influence salivary protein composition [53]. Thus, variations in individual salivary protein compositions between cancer and healthy subjects could indirectly influence the post-rinse recovery of Fe from saliva.

## Conclusions

For the malignant glioma patients in this study, the results did not show trends in salivary lipid oxidation, total proteins, or levels of individual metals in saliva over the course of their 30-week cancer treatment. These three salivary parameters also did not provide for reliable measures of chemosensory dysfunctions over time. When compared with the saliva of healthy subjects, the malignant glioma patients had significantly higher levels of salivary lipid oxidation, total protein, and Na, Cl, K, Mg, P, and S; patients had statistically lower levels of salivary Fe and Zn. Implications of this study are that the differences in salivary constituents for malignant glioma patients should be further explored to find their causes and that further research into sources of chemosensory dysfunction are warranted.

Additionally, our findings identify a need to distinguish and quantify chemosensory dysfunction in cancer patients based on sensitivity to basic tastes and metallic stimuli, as the latter is dominated by retronasal smell rather than taste functions.
